# Developing a Complex Educational–Behavioural Intervention: The TREAT Intervention for Patients with Atrial Fibrillation

**DOI:** 10.3390/healthcare4010010

**Published:** 2016-01-14

**Authors:** Danielle E. Clarkesmith, Helen M. Pattison, Christian Borg Xuereb, Deirdre A. Lane

**Affiliations:** 1 School of Health and Life Sciences, Aston University, Birmingham B4 7ET, UK; danielleesmesmith@hotmail.com (D.E.C.); h.m.pattison@aston.ac.uk (H.M.P.); christianborgxuereb@gmail.com (C.B.X.); 2 University of Birmingham Institute of Cardiovascular Sciences, City Hospital, Birmingham B18 7QH, UK; 3 Department of Psychology, University of Malta, Msida MSD 2080, Malta

**Keywords:** common-sense model, intervention, necessity-concerns, atrial fibrillation, anticoagulation

## Abstract

This article describes the theoretical and pragmatic development of a patient-centred intervention for patients with atrial fibrillation (AF). Theoretical models (Common Sense Model, Necessity-Concerns Framework), clinical frameworks, and AF patient feedback contributed to the design of a one-off hour-long behaviour-change intervention package. Intervention materials consisted of a DVD, educational booklet, diary and worksheet, which were patient-centred and easy to administer. The intervention was evaluated within a randomised controlled trial. Several “active theoretical ingredients” were identified (for e.g., where patients believed their medication was less harmful they spent more time within the therapeutic range (TTR), with general harm scores predicting TTR at 6 months). Allowing for social comparison and adopting behaviour change techniques enabled accurate patient understanding of their condition and medication. The process of developing the intervention using theory-derived content and evaluation tools allowed a greater understanding of the mechanisms by which this intervention was successful. Alleviating concerns about treatment medication by educating patients can help to improve adherence. This process of intervention development could be adopted for a range of chronic illnesses and treatments. Critical elements should include the use of: (1) clinical guidelines; (2) appropriate theoretical models; (3) patient input; and (4) appropriate evaluation tools.

## 1. Introduction

Complex interventions aiming to change health behaviour contain several interacting components. They may target numerous factors such as patient perceptions, diet, lifestyle, cognitive capacity, physical capability, understanding, knowledge and the behavioural skill-set to perform the required health behaviour [1]. These types of interventions are widely used within public health and clinical practice yet their design and evaluation have been criticised as using a “black box” approach, whereby we are unable to determine how and why interventions work due to incomplete reporting and documentation of process [2]. The Medical Research Council (MRC) framework for the development and evaluation of complex interventions includes pre-clinical and theoretical development [3]. However, the literature surrounding intervention development is still very limited, thus whilst trials report outcomes of their evaluative process, little is known about the mechanisms of successful interventions.

**Figure 1:**
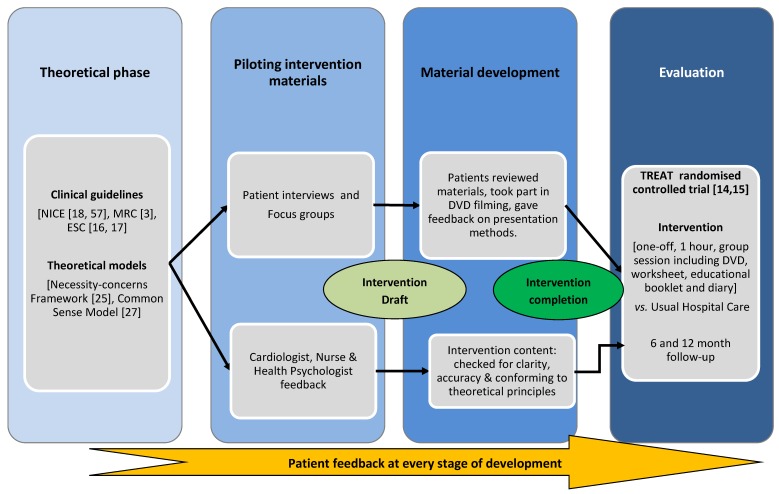
Intervention development process.

A recent Cochrane systematic review suggested the need for a comprehensive behavioural intervention for patients with atrial fibrillation (AF) [4]. This patient group, with a high risk of stroke, has received little attention regarding theory-based intervention development. Patients with AF are prescribed oral anticoagulation (OAC) to reduce their risk of stroke, and their adherence to treatment and recommendations can be objectively monitored over time [5]. This group is in particular need of theoretically-driven educational materials as evidence suggests they have little knowledge of their condition or treatment [6,7,8], poor adherence to medication and associated lifestyle recommendations [9,10], high levels of anxiety and depression [11,12] and inaccurate illness representations [13].

This article will describe the theoretical and pragmatic development of a patient-centred intervention for patients with atrial fibrillation (AF). The process undertaken to design and develop the TRial of an Educational Intervention for patients with ATrial Fibrillation (TREAT) (ISRCTN93952605) [14] intervention will be used as an example of the intervention design and evaluation process. The TREAT intervention has recently been tested in a randomised controlled trial. The main results have been reported [15].

### 1.1. Intervention Development Process

The development of the intervention was a collaborative process involving health psychologists, cardiologists, nurses and patients. Clinical guidelines [3,16,17,18] and theoretical models [19,20] provided the basic content for the educational materials. A parallel patient feedback process ensured the final intervention was patient-centred (see Figure 1). The development process was documented at all stages to ensure it was explicit and replicable.

### 1.2. Theoretical Approach

#### 1.2.1. Application of the Necessity-Concerns Framework

Patient’s beliefs about their prescribed medication can play an important role in the decision to adhere to the required medication regimen and associated lifestyle recommendations. The “Necessity-Concerns Framework” suggests that patients hold beliefs about medication, including its necessity and concerns about dependence and long-term toxicity. It also describes general beliefs about medication, such as its potential to be addictive, harmful, and over-prescribed by doctors. These beliefs, and the way in which patients balance their concern about medications with their beliefs about the necessity for the medication to control their condition, have been widely used in predicting adherence in a variety of chronic conditions including rheumatoid arthritis [21], asthma [22], type II diabetes [23] and depression [24].

Adherence to medication can be both intentional and unintentional. When unintentional, non-adherence can refer to a person’s ability to take their tablets, which is often due to memory impairment. This can be rectified by providing memory aids, dosettes and daily routines (Table 1). However, patients who intentionally choose not to take their medication based on individual motivations and/or beliefs may require a more complex intervention. Patients may report either intentional or unintentional non-adherence or both, and indeed the two concepts may overlap (e.g., if an individual believes medication is unnecessary, they may be less likely to remember to take it) [25]. The necessity-concerns framework has also been found to predict both intentional and non-intentional adherence in patients starting medication for a range of chronic illnesses [25]. 

Cardiac patients are particularly at risk in comparison to oncology or dialysis patients and they are significantly more likely to perceive that the personal costs of their medication outweigh the benefits [26]. When assessing medication benefits patients often consider whether their medication is having an impact upon their symptoms [20]; since cardiac patients often present as asymptomatic, they may not perceive their medication as a “necessity”. By emphasising the importance of, and the benefits of, medication to cardiac, or in this case specifically to atrial fibrillation, patients, it may be possible to increase adherence. Further it seems pertinent to address any misconceptions surrounding the potential harm of the medication prescribed. In order to improve adherence rates, patients must understand the need for treatment and any concerns they have about taking it must be tackled. The intervention must incorporate the needs and concerns of both unintentional and intentional non-adherers.

**Table 1:** Key recommendations regarding beliefs about medications.

Key Recommendations
Address unintentional non-adherence by using reminders/memory aids/dosettes Address intentional non-adherence by discussing with the patient their individual barriers to adherence and the benefits of their prescribed medication Discuss with patients the consequences of their illness, risks associated with treatment and how this related to their perceived barriers to medication adherence

#### 1.2.2. Application of the Common Sense Model

The “common sense model” (CSM) of illness perceptions suggests that patients’ appraisal of somatic changes, such as symptoms, can explain both patient care seeking and management of their condition [25,26,27,28]. For example, symptoms of illness may be attributed to ageing [29,30] or psychological stress [31], thus not prompting treatment-seeking behaviours. Many patients may hold medically unsupported beliefs about their medical condition, derived from the media, extreme cases, lay-persons, or general beliefs about health and illness. The CSM suggests that patients rely on a set of ‘mental tools’ to understand somatic stimuli such as duration, location and severity of symptoms. For example, hypertension patients new to treatment were likely to drop-out of the regimen if they construed the treatment as acute. However, those patients continuing treatment were more likely to believe their medication was having beneficial effects upon their symptoms [20]. In adolescent patients with type 1 diabetes, a change in perceived effectiveness of the diabetes treatment regimen predicted dietary self-care [32]. Thus the patients’ beliefs about their illness and treatment regimen can impact upon their uptake and maintenance of health behaviours. By understanding patients’ illness representations we are able to understand how patients self-regulate their illness and their treatment. This model has been used to predict treatment seeking, medication adherence and patients’ attribution of symptoms [33,34], and has also informed successful interventions to aid management of chronic illnesses [35,36].

The CSM describes five key components of patient illness representations: (i) *identity*—symptoms associated with the specific illness and label ascribed to the illness; (ii) *consequences—*expected physical, social and economic implications; (iii) *timeline*—acute, chronic or cyclical duration; (iv) *causes—*personal beliefs about causes; and (v) *cure/control*—the extent to which a patient believes they will recover from or control their illness [26,27,37,38]. McAndrew and colleagues focused on the use of CSM for intervention design and suggested integrating the five components into an intervention and monitoring the change between current status and the desired endpoint(s) [39]. 

Patients’ common sense models of their physical health and illness can be influenced by subjective cognitions (particularly symptoms, moods, and experienced dysfunction). This symptom-focused prototype can interfere with the successful management of their condition, particularly when patients are asymptomatic, as they may use subjective cues (such as symptoms) to *identify* the illness. For AF patients symptoms may come and go, or not occur at all, giving patients an inaccurate perception of their illness identity. Thus it is important to provide patients with a top-down conceptual framework for AF so that they can recognise that it is present even if they are asymptomatic (see Table 2). 

An additional intervention component which replaces automatic control with volitional control is needed within the intervention. This involves patients knowing what to do to minimise the risk, thus controlling their health outcomes (e.g., dietary change, reducing alcohol intake and use of anticoagulants) and relying on objective rather than subjective indicators (*identity*, using INR as a meter for control, rather than symptoms) to evaluate the efficacy of treatment (*control* and *consequences)* [28,39]. This approach focuses on behavioural change by using an action plan and/or diary. The volitional control system can then be integrated into daily behaviour and will eventually become automatic [39].

**Table 2:** Summary of the requirements for patients to create a cognitive representation of AF.

**Create an illness identity**	Help patients understand which symptoms are/are not associated with AF, common co-morbidities, the risks of stroke and the reasons for prescribing anticoagulant medication and the emotions individuals associate with the illness (e.g., “I am afraid of what will happen“).
**Understand the consequences**	Help patients understand the physical, social and economic implications of both AF and treatment with anticoagulation. Patients need to be provided with information about the risks associated with AF e.g., the main risk associated with AF is stroke.
**Identifying their illness timeline**	Patients can be made aware of the duration of their illness and treatment and given information about the different types of AF and how this relates to the risk of stroke.
**Understanding the causes**	Patients need to recognise their personal ideas about the causes of AF and how they relate to the scientific evidence.
**Identifying a cure or control for their illness/symptoms**	Patients can be presented with information pertaining to the control of their INR and pharmacological control of their AF symptoms, and explore the factors that may affect their symptoms including caffeine intake, exercise and alcohol. Of particular relevance are the key lifestyle factors that affect INR control including diet, alcohol intake and other medications and supplements, as for many patients there is no “cure” for AF.

By eliciting concerns about treatment and prescribing a convenient treatment regimen tailored to address practical barriers to adherence, patients may be more likely to manage their illness effectively [39]. Patients are taught to become expert self-managers, formulating an action plan to monitor their intake of foods that are high in vitamin K and thus affect INR control, alcohol intake, and other medications and supplements. Generally INR monitoring is largely hospital-based and thus not truly a self-monitoring process, however self-monitoring/self-management of warfarin is a viable option for selected patients. Hence whilst patients can still develop action plans, they are not able to use INR as an objective self-monitoring indicator. However, patients can reflect upon lifestyle factors that influence their INR control by using a self-monitoring diary. This acts as a feedback-loop, allowing patients bottom-up inputs (INR test results) to act as a monitor for control. Monitoring is often reduced as the INR stabilises after the first few weeks, thus the diary can be used for the initial few weeks of the treatment. Furthermore patients can create their own action plan specifically focusing on the areas that they personally need to control, for example, some patients may feel their current alcohol, dietary intake or vitamin supplements could cause problems for their INR control.

## 2. Experimental Section

### 2.1. Development of the Intervention Materials

The design of this intervention was aimed at being as explicit as possible to avoid “unseen” content and to ensure it is replicable within a usual hospital care setting. We have described its components using a taxonomy of behavioural change techniques defined as reliable and theory-derived [40].

### 2.2. Intervention Outline

The one-off, hour-long session was delivered to groups of 1-6 newly-diagnosed, warfarin-naïve patients in a hospital setting. Patients took part in a discussion group; worksheet based activities, and watched a DVD. Patients also took educational materials home including a booklet and self-monitoring INR and lifestyle diary. Each of the intervention components mapped onto the theoretical framework and incorporated several behaviour-change techniques which are detailed further in Table 3. An example of a behaviour change technique developed is to “provide information on consequences”. By providing patients with the educational information about stroke risk and treatment risks (such as bleeding), patients are able to create their own (accurate) perception of their illness and its potential consequences. When completing the worksheet patients use a stroke risk acronym (CHADS_2_ [41] score at the time of the intervention development (based on clinical guidelines in 2009 [42]) but now the CHA_2_DS_2_-VASc score [43] would be used based on current clinical guidelines [16,17,18]) to calculate their own risk of stroke, they also watch a DVD of other patients discussing their own experiences of treatment (what happens if you adhere/do not adhere) and they discuss the consequences of their illness and their experiences of those consequences (for example, transient ischemic attack or stroke). Thus we used different methods (including DVD, worksheet, and booklet) to provide education, ensuring the message was accessible. Table 3 documents the educational components of the intervention and how these relate to theoretical models and behaviour change techniques.

### 2.3. Patient Involvement

Once the theoretical basis for intervention development was established and appropriate educational information had been gathered (based on clinical guidelines published by the National Institute for Health and Care Excellence (NICE) [18], various presentation methods, such as detailed explanations, diagrams, graphs, pie chart and pictograms (as derived from [44] were piloted with AF patient groups. The key objective of the implementation study was to establish AF patients’ preferred communication methods for the educational information and to ensure the final materials were patient-focussed and could be easily understood (using the “teach-back” method). Patients were asked to relay the key message that the information was trying to convey, or to explain the information to the researcher and/or the other participants. For this purpose two focus groups and six individual interviews were carried out. The first focus group was exploratory, drawing on patient’s experiences and perceptions of their illness and treatment and helped to inform the content of the educational materials (booklet, DVD and patient narratives). This paper will draw on the second focus group and the interview data. During this focus group, 6 warfarin-experienced patients with AF (3 males and 3 females) were presented with information slides; the same information was also presented to 4 patients who were new to warfarin, within the interviews. During the focus groups patients came to a consensus regarding which information and presentation styles to include. For the purpose of this development paper, we will draw on some examples that illustrate patient involvement in the intervention design. 

#### 2.3.1. Description of Symptoms 

Patients were presented with a slide that described the symptoms associated with AF, taken from a published article outlining associated symptoms [45]. They were asked whether this clearly described the symptoms they attributed to their AF. Two key factors were raised. Firstly, some patients are asymptomatic and felt this should be clearly stated within the materials. One male AF patient stated: *[M2]: “I can only say that on the last article on….other people with atrial fibrillation, I had no symptoms at all and it came about by a routine inspection and I believe that I told... when I say... symptoms I am talking about the chest discomfort, the light headedness, the tiredness, or fainting... none of that”.*

Secondly, for several patients their symptoms had previously been misattributed to other causes and a clear list of symptoms clarified the identity of their illness, one female patient explains [*F1*]: *“when I first started experiencing palpitations and tiredness and slight shortage of breath... occasionally... I was told that it was all part of erm... the work I was doing at the time and it was an occupational hazard.”* Thus, a clear list of symptoms is included in the intervention materials, noting that some patients may also be asymptomatic.

**Table 3:** Theoretical intervention components.

Behavioural Change Technique	Method of Delivery	Intervention Components	Theoretical Model Targets
**Provide general information on behaviour-health link**	DVD (AF patients, cardiologist) Booklet	Education on AF and OAC Advice on alcohol intake, diet and other medications and supplements Risk information for stroke (with and without medication) Risks of non-adherence	Necessity-concerns Illness perceptions (Consequences, timeline, causes)
**Provide information on consequences**	DVD (AF patient experiences of bleeding/bruising) Worksheet (action plan, risk calculations) Booklet (risk of stroke/side effects percentages)	Calculate their own risk of stroke using the stroke risk tool Draft action plans to minimise this risk Presentation of risk of stroke and bleeding complications	Illness perceptions (identity) Necessity-concerns Illness perceptions (Consequences)
**Prompt barrier identification**	Group discussion Worksheet questions	Think about potential barriers to adhering to recommendations Design a personal action plan to overcome these barriers Discuss barriers that other patients have identified and the ways in which they made changes to overcome these barriers List the 3 main concerns about taking warfarin; raise these concerns within the group discussion, or talk to the researcher following the session	Necessity-concerns Illness perceptions (identity)
**Provide instruction**	DVD (Cardiologist) DVD (mock consultation) Booklet	Informing the patient how to perform specific health behaviours such as ‘what do if you miss a dose of anticoagulation’, ‘how to remember to take your tablets’, and ‘when to seek medical attention’ Formulate a personal action plan which will include memory aids for medication and what to do in an emergency	Unintentional non-adherence
**Prompt self-monitoring of behaviour**	Self-monitoring diary Worksheet (Action plan)	Keep a daily record of dietary intake, alcohol units and medications and supplements for a 2-week period Self-reflective tool to encourage patients to assess what lifestyle changes may have had an impact on their INR values	Intentional non-adherence Necessity-concerns Illness perception (Consequences & control)
**Teach to use prompts/cues**	DVD (AF patients) Worksheet plan	Patients are encouraged to identify environmental prompts which can be used to remind them to take their medication Group discussion of the roles of their partner or family members within their treatment regimen. Patients are encouraged to discuss their own memory-aid methods	Intentional/unintentional non-adherence
**Provide opportunities for social comparison**	DVD (AF patients) Group discussion	Delivery of intervention in a group setting allows for social comparison Patient narratives from existing AF patients taking warfarin, using the DVD	Necessity-concerns (unintentional-non adherence) Illness perception (Causes, controls and consequences)

#### 2.3.2. Types of AF

Patients were presented with definitions of the different types of AF (see Box 1), they discussed their understanding of the definitions, which category they fitted into and why.

Box 1 **Classification of atrial fibrillation [18].**

1. **Paroxysmal**: multiple episodes that typically last less than 48 h and stop by themselves.
2. **Persistent:** episodes that last longer than 7 days, or stop when treated.
3. **Permanent:** continuous atrial fibrillation for more than 1 year.

All of the patients appeared to be able to quickly categorise themselves (see dialogue below in (Box 2), thus the descriptions were kept the same.

Box 2 **Patient quotes surrounding definitions of AF.**

*[M2]:*  “out of that... number 3 will be the nearest to me... permanent continuous atrial fibrillation... for more than 1 year and I would say 50 years... that's how long I can go back... I can only say its number 3 for me because of the length of time”.
*I* –  “how about everybody else?”
*[F1]: *  “I have spent 3 years since I was diagnosed with it. With atrial fibrillation”
*I*–  “and which category would you fall into?”
*[F1:]*  *“mmm I would say probably the first one... comes and goes...”*

#### 2.3.3. Risk Presentation

Patients were shown three different presentation methods for the same risk information. The information related to the risks of stroke associated with AF and the risks of bleeding associated with treatment with warfarin. Presentation methods included two traditional methods: pie charts, bar charts and one more novel method, pictograms (denoting each percentage as a smiley face, previously used as a decision making tool [44,46]). Patients were asked which method they preferred and which they understood. All patients agreed that the pie chart presentation method was clearer for a range of statistical risk information. One patient described how time-consuming the pictogram method was [*F1]: “because otherwise you are going to be sitting there ages as I say…counting how many faces are there on there…whereas with the circle you got…straight away”.* The pie chart presentation was adopted for the presentation of risk information within the educational booklet (see Supplementary Figures).

#### 2.3.4. Diagram of the Heart and Formation of Clots

Patients were presented with a diagram (of a torso and diagram of the heart, nervous system and brain) describing the formation of clots within the left atrium (top chamber) of the heart and the subsequent risk of stroke. They were asked whether the diagram was useful and whether they understood information they were presented with. The dialogue indicated that this diagram provided the informational link between the risk of blood clots and the risk of stroke (see Box 3).

Box 3 **Patient quotes surrounding stroke torso diagram.**

*[F1]:*  *“well its explaining what can happen in the sections of the heart and how erm, as it there, clots can form and erm”*
*[M1]:*  “go to the brain”
*[F1]: *  “yeah… can go to the brain and that can cause erm strokes or whatever and at the same time, it is also showing how the different movements of the heart, the pumping of the heart”
*[M1]:*  “oh yes”
*[F1]:*  “can effect erm… this distribution shall we say unless it is controlled with a thinning… drug or whatever. That’s how I look at it”

Another patient described how the information provides an explanation for the need for anticoagulation with warfarin [F2]- “it would help and it would help you to know why you have been given your medication and what it was gonna do to help prevent you… you know having the blood clot in first place”. The original diagram was digitalised and adapted for use within the educational booklet.

## 3. Results

### 3.1. Evaluation of the Intervention

The intervention was evaluated in a randomised controlled trial comparing patients receiving the intervention in addition to usual care (attendance at the hospital OAC clinic) with usual care alone. The protocol for this trial has been published elsewhere [14], the trial is registered with Current Controlled Trials [47] and the trial design is summarised in Table 4. There were no significant differences between the groups at baseline on any of the demographic variables.

**Table 4:** Summary of the TREAT randomised controlled trial.

Trial No	ISRCTN93952605
**Target group**	**N Randomised:** 46 intervention *vs.* 51 usual care **Diagnosis:** Warfarin-naive AF patients **Demographics of cohort:** Mean (SD) age 72.9 (8.2) years; 64.9% male; 99% White British, Irish or European. No significant differences in demographic variable between groups. **Inclusion/exclusion criteria:** AF patients newly referred for warfarin therapy, with ECG-documented AF, will be eligible for inclusion. Patients were excluded if they were aged <18 years old, had any contraindication to warfarin, had previously received warfarin, had valvular heart disease, cognitively impaired, unable to speak or read English or had any disease likely to cause their death within 12 months.
**Intervention**	**Type: **One-off group [1–6 patients] theory-driven educational intervention Content: DVD, educational booklet, worksheet, group discussion. **Duration:** 1 h session **Facilitator:** Health-Psychologist (could be delivered by trained lay educator) **Setting:** Hospital outpatients clinic
**Outcomes**	**Primary outcome:** Time within therapeutic range (TTR) calculated using the Rosendaal method **Secondary outcomes:** Patient knowledge, Beliefs about Medication, Quality of Life, Anxiety and Depression, Hospital Admissions and Adverse events.
**Comparison group**	Usual Hospital Care
**Random sequence generation**	A computer generated list stratified by (a) age (<70 and ≥70 years)/sex and (b) specialist AF clinic *versus* ‘general’ cardiology clinic, in blocks of four, randomised patients on an individual basis to receive either ‘usual care’ or the intensive educational intervention, in addition to ‘usual care’. The randomisation schedule was designed by an independent trials unit.
**Blinding**	A researcher not involved in the data analysis or intervention delivery matched patient ID numbers with randomisation codes and checked follow-up questionnaires for completeness. The researcher analysing the data was blinded to which arm of the intervention patients were randomised to.

The findings of the randomised controlled trial which tested the TREAT intervention have been published elsewhere [15]. In summary, the intervention group had a significantly higher proportion of time in the therapeutic range (TTR) (a measure of anticoagulation control and medication regimen adherence) at 6 months than the usual care group (76.2% *vs.* 73.1%; *p =* 0.035); these differences were not significant at 12 months (76.0 *vs.* 70.0%, *p =* 0.44). However it is important to consider why and how this intervention may have proved successful in its primary objective of improving TTR. The two theories that formed the basis of the intervention development were the beliefs about medication theory and the common sense model for illness perceptions [20,27]. 

### 3.2. Beliefs about Medication

There were significant differences between intervention and usual care groups perception of medication harm (F (1, 28) = 4.4; *p <* 0.05), the intervention group viewed the intervention as less harmful than the usual care group [15]. There were no significant differences between groups on the other subscales.

There was also a significant interaction between groups across time for perceived medication harm (F (4, 28) = 2.4; *p =* 0.04), perceived general overuse of medication (F (4, 28) = 2.4, *p =* 0.04) and concerns about medication (F (4, 27) = 2.9, *p =* 0.02), but no interaction for perceived necessity of medication. There were no significant changes in scores on the subscales across time. The general harm scores at 1 month was the only significant predictor of the 6-month TTR scores (F (1, 72) = 4.08, *p =* 0.04) [15]. 

### 3.3. Illness Perceptions

Patients’ perception of the AF timeline (acute, chronic or cyclical) differed significantly across time (F (4, 25) = 5.9; *p <* 0.01), but not between groups. Perceived treatment control (F (4, 25) = 2.7; *p =* 0.05), emotional representation (F (4, 26) = 3.1; *p =* 0.04), and illness coherence scores changed significantly over time (F (4, 26) = 4.6; *p <* 0.01), but not between groups. IPQ factors did not predict TTR at 6 or 12 months [15]. 

There were no significant differences between groups across any of the illness perception questions other than perceived cause (see Table 5). At baseline, the majority of patients thought that there was a psychological cause (e.g., stress or bereavement; 41%), or an external cause of their illness (e.g., age or previous morbidity; 31%); fewer blamed lifestyle factors (e.g., smoking, diet or lack of exercise; 29%). At the one month follow-up, more patients believed that there was an external cause of their AF (48%), rather than a psychological cause (33%). At each follow-up, the intervention group was more likely to perceive the cause as external, while patients in the usual care group were more likely to perceive the cause of their AF as psychological. These differences were significant at 6 months (*p =* 0.04). This indicates that patients did change their perception of cause after receiving the intervention. 

## 4. Discussion

The results from this study give some insight into the mechanisms by which the intervention has proved successful in improving patient INR control. Where patients viewed their medication as less harmful, they were more likely to adhere to their medication, as reflected by their TTR scores, a finding which supports previous evidence [25,30,31]. Thus patient’s perception of the ”toxicity“ of medications, and their views surrounding the impact of side effects in the long- and short-term may have changed as a result of the intervention. However, intervention design seldom considers the impact that medications perceived by the patient as harmful will understandably have on medication adherence. 

When deciding whether or not to initiate new medication a patient undertakes an individual risk-benefit assessment [32], this process is important as treatment choices carry significant risks to patient safety if the guidelines and lifestyle recommendations are not adhered to. Their ability to do this is dependent on assimilating information about the risk of stroke conferred by AF against the risk of bleeding associated with warfarin, a process which cannot take place accurately without appropriate education and delivery of information (educational tools).

Both groups scored highly on the specific-necessity subscale at all time-points, suggesting that all patients within the trial viewed their medication for AF as highly necessary. However, patients in the intervention group were less concerned about their medication at all time-points. The greater difference between necessity and concerns scores in the intervention group, suggests patients perceptions about the necessity for warfarin outweighed their concerns about taking it. Evidently AF patients view the reduction of stroke risk, and subsequently the need for treatment, as more important than the potential bleeding risks associated with warfarin. This also supports evidence surrounding patients’ perceptions of warfarin and their willingness to accept bleeding risks in order to reduce their risks of suffering from a stroke [46,48,49]. 

The present findings suggest that there were no significant differences between groups across any of the illness perception questions other than perceived cause. This contradicts previous evidence where interventions using the principles of the common sense model have found changes in illness perceptions [35,36]. However, previous studies have used a more individualised intervention approach; assessing unique illness perceptions, and targeting specific problematic areas [35,36]. Thus, the TREAT intervention, which aims to be accessible for the lay educator within usual hospital care, and generic in its approach, may not have the same impact, but rather help to prevent the formation of inaccurate illness representations. 

Patients in the intervention group had a more accurate perception of their illness following the intervention, stating established causal factors including age, male gender, hypertension, valvular heart disease, heart failure, coronary artery disease, diabetes, [16,17,18,50,51] and genetic factors [52,53]. AF patients have previously reported psychological causes of AF [13], despite evidence to the contrary; which could contribute to the emotional burden of their illness. Changing lifestyle factors represents an area patients are able to control, as opposed to external factors such as age, or other co-morbidities. This change in perceived causality may have impacted upon the patients’ perceived control over their illness and treatment. 

Using the IPQ-B as an evaluation tool has its limitations. It does not assess patients’ perception of the cyclic nature of their illness. Previous evidence suggests AF patients view their illness as cyclic rather than long-term [13]. However, McCabe and colleagues had a higher percentage of symptomatic paroxysmal patients (64%) in their study, thus the results may be affected by the differences between samples. The validity of the IPQ-B has been deliberated in a recent article [54]. Thus, it is possible that some of the differences between groups were not identified due to poor sensitivity of this tool.

Patients in the intervention group achieved a significantly greater proportion of TTR after 6-months of warfarin than patients receiving usual care alone [15]. However, 12-months after initiating warfarin there was no significant difference in TTR between the groups although TTR was higher in the intervention group [15]. Therefore, patients with chronic conditions which require life-long adherence to medication and lifestyle modification, as with AF, may require interventions such as TREAT to be repeated at regular intervals in order to refresh information and reinforce behavioural changes. 

Whilst patients are often encouraged to adhere to treatment recommendations, general practitioners and physicians rarely discuss the behaviours required to become adherent and perceived treatment barriers [55,56], thus interventions are not tailored to patient’s needs. The TREAT intervention was a collaborative project between patients, the theoretical approaches of health psychologists, and the clinical guidance of cardiologists and existing clinical guidelines [3,16,17,18,57]. The successful management of chronic illness is a multidisciplinary process, which integrates the patient and their beliefs into every aspect of clinical care. We hope that by documenting this process, other researchers will benefit in the future when designing complex interventions. 

**Table 5:** Patients’ perceived cause of atrial fibrillation.

N (%)	Baseline		χ^2^	1 Month		χ^2^	2 Months		χ^2^	6 Months		χ^2^
	Intervention (*n =* 25)	Usual care (*n =* 34)		Intervention (*n =* 24)	Usual care (*n =* 24)		Intervention (*n =* 19)	Usual care (*n =* 19)		Intervention (*n =* 23)	Usual care (*n =* 21)	
Psychological	10 (40.0)	14 (41.2)	1.38	8 (33.3)	8 (33.3)	0.15	3 (15.8)	9 (47.4)	4.47	3 (13.0)	9 (42.9)	6.31 *
External	6 (24.0)	12 (35.3)		11 (45.8)	12 (50.0)		12 (63.2)	8 (42.1)		17 (73.9)	8 (38.1)	
Lifestyle	9 (36.0)	8 (23.5)		5 (20.8)	4 (16.7)		4 (21.1)	2 (10.5)		3 (!3.0)	4 (19.0)	

* *p <* 0.05; psychological: includes answers relating to psychological morbidity including stress, anxiety, depression and bereavement; lifestyle: includes factors related to patients lifestyle habits such as smoking, excess drinking, obesity and excessive exercise; externals: includes factors that patients cannot control including hereditary disposition, following surgery or other chronic illness.

## 5. Conclusions

Complex interventions for use with patient health behaviours need to be theory driven and based on relevant current literature and clinical guidelines. The success of an intervention relies on patient involvement. The TREAT intervention changed patient beliefs surrounding their treatment necessity and treatment harm. We used several behavioural-change techniques to relay these messages, but the most powerful messages were the lived experiences of the AF patients themselves, presented as narratives on the DVD. As healthcare professionals we cannot assume patients understand the necessity of their medication and we cannot underestimate the impact of individual perceptions of medication and illness on the management of a chronic illness.

### Practice Implications

Future researchers and healthcare workers aiming to develop patient interventions need to be explicit about the content and theoretical components to enable replication of successful interventions. Interventions for patients with AF starting warfarin should include the following key elements to enable behaviour change:

- Provide educational materials and discuss the health-behaviour link, enabling patients to understand why and how they make lifestyle changes.
- Provide educational materials and risk information on the consequences of AF and treatment with/or without warfarin
- Provide opportunities for social comparison with other patients
- Encourage patients to self-monitor, create action plans, and use their own memory aids/cues for remembering to take tablets
- Discuss patients concerns and barriers to changing their lifestyle and adopting a new treatment regime; correct any misconceptions with accurate information.

Presenting the full methodology behind the development of interventions could enable intervention design to become a much more efficient and transparent process.

## Supplementary Materials

Supplementary Figures S1–S4 are available online at http://www.mdpi.com/2227-9032/4/1/10/s1.
