# Supplementary material for: Developing a Complex Educational–Behavioural Intervention: The TREAT Intervention for Patients with Atrial Fibrillation
Source: Healthcare (Basel). 2016 Jan 14;4(1):10. doi: 10.3390/healthcare4010010 (PMC4934544; doi:10.3390/healthcare4010010)
Supplement: Supplementary file 1 [file healthcare-04-00010-s001.pdf]

Supplementary Materials: Developing a Complex Educational-Behavioural Intervention: The TREAT Intervention for Patients with Atrial Fibrillation

Danielle E. Clarkesmith, Helen M. Pattison, Christian Borg Xuereb and Deirdre A. Lane

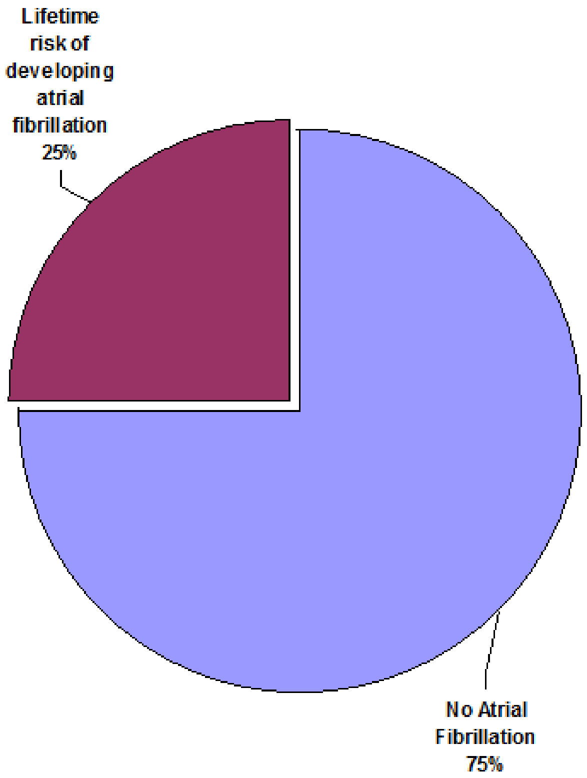

Figure S1. There is around a 25% lifetime risk of developing atrial fibrillation for people aged 40 years and older. The risk is the same for men and women.

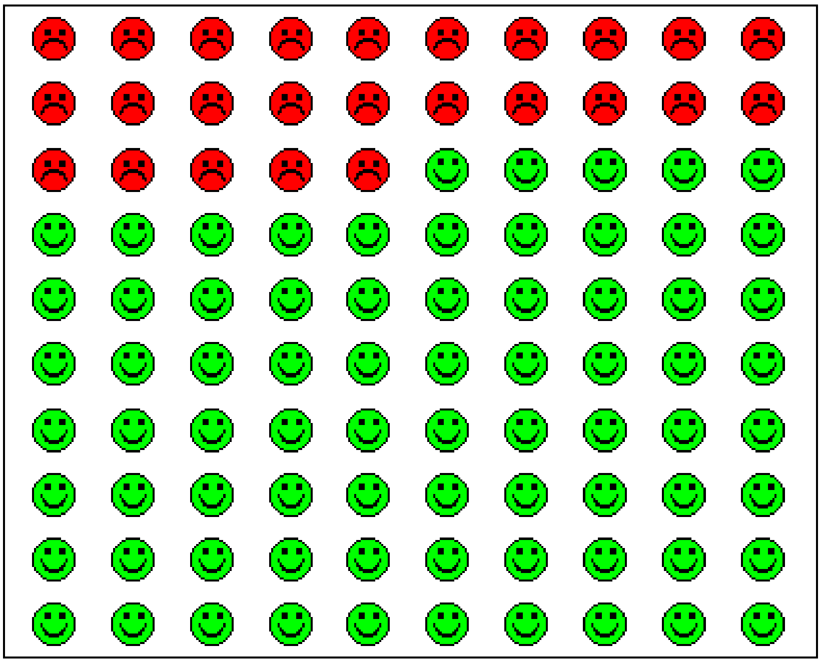

Figure S2. There is around a 25% lifetime risk of developing atrial fibrillation for people aged 40 years and older. The risk is the same for men and women.

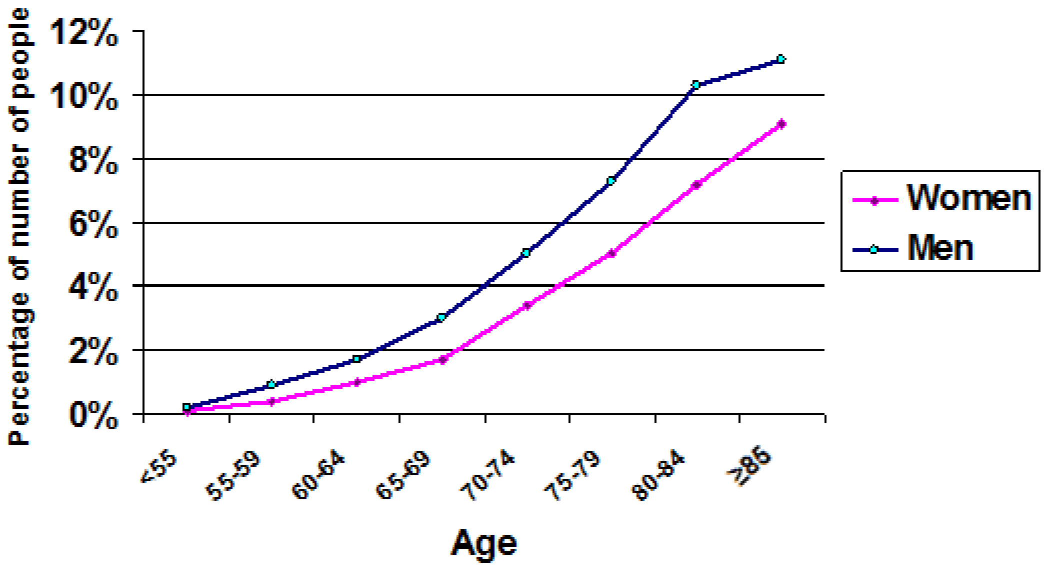

Figure S3. Increase in the number of people with Atrial Fibrillation as age increases.

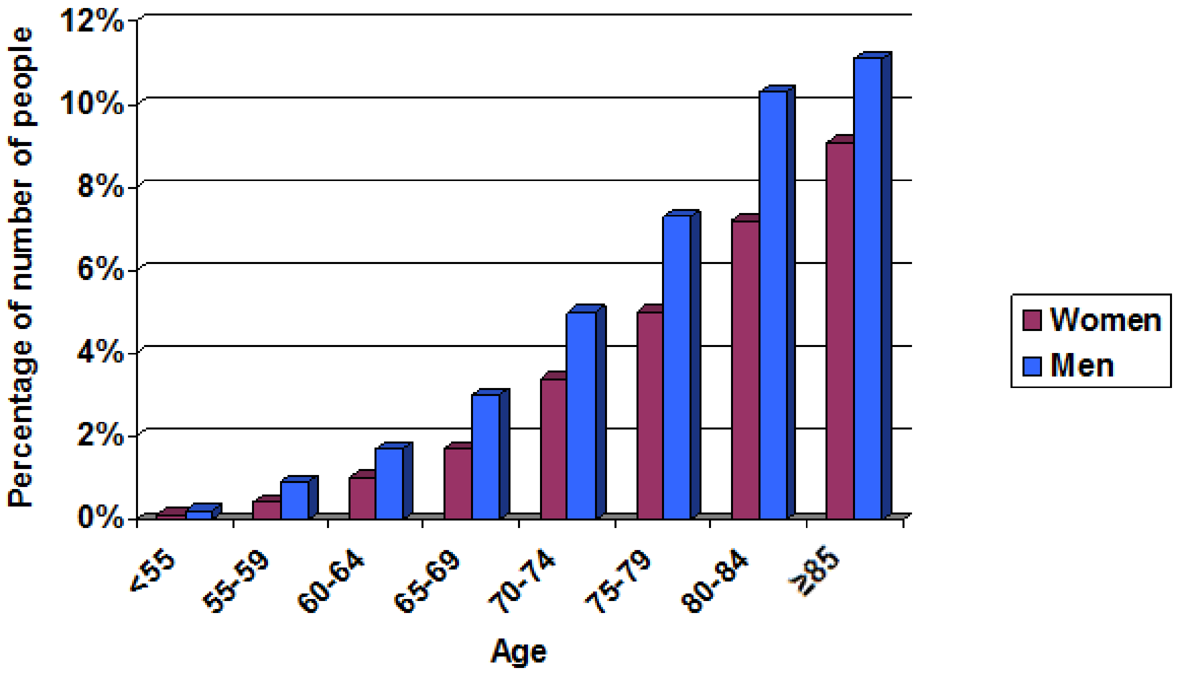

Figure S4. Increase in the number of people with Atrial Fibrillation as age increases.

Patients were shown different methods of presenting risk information. For example, Supplementary Figures 1 and 2 relate to the lifetime risk of developing atrial fibrillation in people aged 40 years and older using a pie-chart and a pictogram. Supplementary Figures 3 and 4 demonstrate the increasing risk of atrial fibrillation as age increases using a line graph and a bar chart. For each piece of information patients were asked which method they preferred and which they understood and they were asked to explain what each Figure represented. All patients agreed that the pie chart presentation method was clearer for a range of statistical risk information because it was visually easy to comprehend whereas the pictogram was more difficult and time-consuming to understand (counting the different coloured faces) and therefore the pie chart presentation was adopted for the presentation of risk information within the educational booklet. Patients also felt that the bar graph was a better way of presenting the increasing risk of atrial fibrillation with age than the line graph.
